# Ultrasonography of the Kidneys in Healthy and Diseased Camels (Camelus dromedarius)

**DOI:** 10.1155/2020/7814927

**Published:** 2020-10-21

**Authors:** Mohamed Tharwat

**Affiliations:** ^1^Department of Veterinary Medicine, College of Agriculture and Veterinary Medicine, Qassim University, P.O. Box 6622, Buraidah, 51452, Saudi Arabia; ^2^Department of Animal Medicine, Faculty of Veterinary Medicine, Zagazig University, 44519, Zagazig, Egypt

## Abstract

This review article is written to describe the results of ultrasonography of the kidneys in healthy camels as well as camels with some renal disorders. In the dromedary camel, the physiology of the kidney is of interest in view of the specialization of the camel to hot dry deserts and to prolonged periods without water. It plays an important role in water conservation through the production of highly concentrated urine that may predispose animal to varieties of renal disorders. Examples of kidney affections in dromedary camels are renal capsular pigmentation, medullary hyperemia, subcapsular calcification, cortical and medullar discoloration, hemorrhage in renal pelvis, nephrolithiasis, and hydatidosis. Congestion, hemorrhage, hydronephrosis, acute glomerulonephritis, subacute glomerulonephritis, chronic glomerulonephritis, diffuse interstitial nephritis, focal interstitial nephritis, renal cyst, hyaline degeneration, renal amyloidosis, tubular nephrosis, pyelonephritis, hemosiderosis, and renal toxicity. When the kidney is examined by ultrasonography, the clinician can get sufficient information about the size, position, and echo patterns of the renal cortex and medulla and renal pelvis and outlines of the renal blood vessels. In recent years, ultrasonography has been used in camels for scanning of the healthy status as well as evaluation and determining the diagnosis and prognosis of diseased cases. Examples of diseases evaluated by ultrasonography are paratuberculosis, trypanosomiasis, pneumonia, pleurisy, gastrointestinal neoplasms, chronic peritonitis, splenic abscessation, and hepatic disorders. Of the renal disorders assessed by ultrasonography are nephrolithiasis, hydronephrosis, pyelonephritis, renal abscessation, and renal neoplasms. Ultrasound guidance in biopsy of renal specimens has also been reported in dromedary camels.

## 1. Introduction

The kidney of the dromedary camel (Camelus dromedarius) plays a vital role in water conservation through the production of highly concentrated urine that may predispose camel to varieties of renal disorders. In an abattoir based study conducted on 100 camels in Najaf-Abad, Iran, the frequency and types of renal affections were determined [1]. These disorders included renal capsular pigmentation, medullary hyperemia, subcapsular calcification, cortical and medullar discoloration, hemorrhage in the renal pelvis, nephrolithiasis, and hydatidosis [1]. Of the later report, capsular melanosis, acute tubular necrosis, chronic interstitial nephritis, caseous necrosis, calcification, medullary hyperemia, and hydatid cyst were confirmed by histopathological examination. In another study conducted in Rajasthan, India, 121 renal samples were examined for various pathological abnormalities [2]. Lesions included congestion, hemorrhage, hydronephrosis, acute glomerulonephritis, subacute glomerulonephritis, chronic glomerulonephritis, diffuse interstitial nephritis, focal interstitial nephritis, renal cyst, hyaline degeneration, renal amyloidosis, tubular nephrosis, pyelonephritis, hemosiderosis, and renal toxicity [2]. In a third study conducted in Al-Ahsa abattoir, Saudi Arabia, gross and microscopic lesions of the kidney were investigated in 50 adult camels [3]. Of the camels, 33 (66%) had gross kidney lesions, whereas 17 (34%) were found apparently healthy. Renal lesions included hydronephrosis, renal hemorrhages, and renal necrosis. In the camels with renal lesions, microscopic changes included glomerular shrinkage and hyalinization, proteinaceous casts, cortical and medullary congestion, tubular cell swelling, interstitial hemorrhage, and thickening of the glomerular tufts [3].

In bovine, thoracic and abdominal ultrasonography is commonly performed to evaluate the type and severity of lesions in animals suspected to have cardiopulmonary, gastrointestinal, hepatic, renal, splenic, and pancreatic disease [4–10]. Therefore, ultrasonography of thoracic and abdominal organs in bovines is a minimally invasive and cost-effective method for early detection of thoracic and abdominal disorders [8, 11, 12].

In camel medicine, ultrasonography was rarely used with the exception in the reproduction field. Recently, in healthy camels, our research group has used ultrasound for scanning of the lungs and pleura [13, 14], echocardiography [14, 15], ultrasonography of the gastrointestinal tract (GIT) [16, 17], hepatic and renal imaging [17, 18], and abdominal ultrasonography [19]. Ultrasound-guided hepatic and renal biopsy and portocentesis have also been carried out in camels [17, 20, 21]. In diseased camels, diagnostic ultrasonography has been done for the evaluation and prognosis of abdominal distension [22], Johne's disease [23], trypanosomiasis [24], abdominal disorders [19, 25, 26], urinary disorders [27], thoracic affections [14, 28], renal neoplasms [29], pyelonephritis and renal abscessation [30, 31], GIT masses [32, 33], chronic peritonitis [34], splenic abscessation [35], and hepatic disorders [17].

The physiology of the kidney of the dromedary camel is of interest in view of the specialization of the camel to hot dry deserts and to prolonged periods without water. In this species, ultrasonographic diagnosis of renal disorders has received lesser attention compared to other animals; thus there is a shortage of information in this area. The procedure has been adopted widely as a diagnostic procedure and research tool in animals. This review article is written to describe the results of ultrasonography of the kidneys in healthy camels as well as camels with some renal disorders.

## 2. Anatomy of the Kidneys in Camels

**Figure 1:**
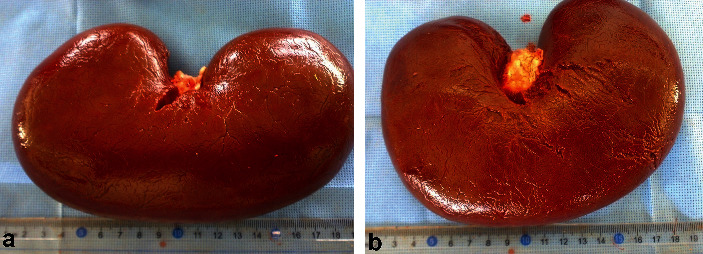
Right (a) and left (b) kidneys in an adult camel (both with removed renal capsules).

**Figure 2:**
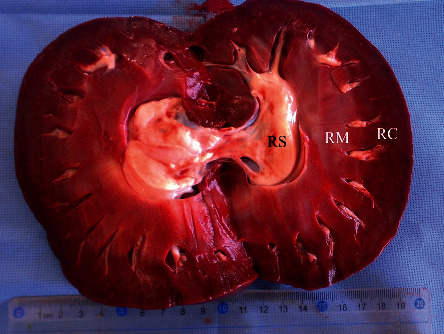
Longitudinal section through the kidney in an adult camel. RC, renal cortex; RM, renal medulla; RS, renal sinus.

**Figure 3:**
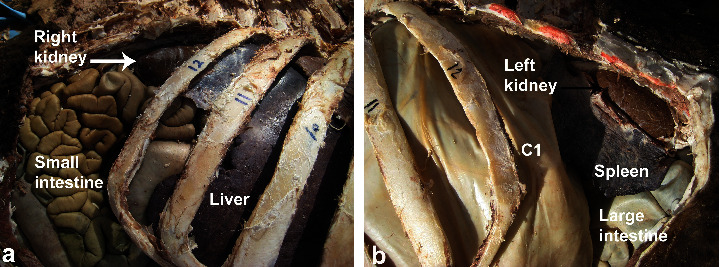
Anatomical position of the right (white arrow) (a) and left (black arrow) (b) kidneys in a camel carcass preserved with 10% formalin solution. *C*1 = first gastric compartment.

The kidneys in camels are bean-shaped and smooth externally. The right kidney is more elongated than the left one, with an average weight of 1.08 kg and 1.13 kg for the right and left kidneys, respectively (Figure 1). On a longitudinal section made through the kidney, the cortex and the medulla are easily distinguished (Figure 2). There is a well-developed *Crista renalis*. The renal cortex occupies about 50% of the volume of the kidney and the ratio of the thickness of the medulla to that of the cortex is approximately 4 : 1. Both kidneys are situated against the dorsal body wall and are retroperitoneal. The right kidney lies below the transverse process of the 2nd to the 4th lumbar vertebrae. Its cranial pole is round and it fits into the renal impression of the caudate lobe of the liver. Its caudal pole is not as rounded and is slightly flattened dorsoventrally. The left kidney is regular in shape and lies below the left transverse processes of the last three lumbar vertebrae [36]. The position of the right and left kidneys in a camel carcass preserved in 10% formalin solution is presented in Figure 3.

## 3. Ultrasonographic Examination of the Kidneys in Camels and Normal Findings

**Figure 4:**
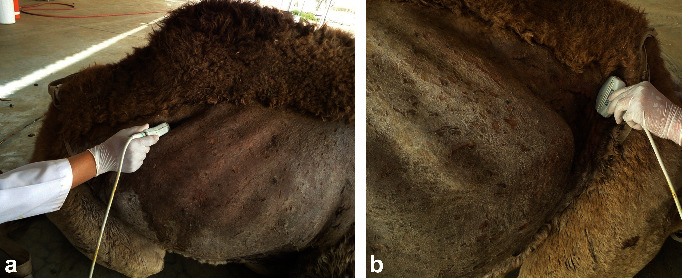
Imaging of the right kidney in the upper right flank (a) and the left (b) kidney in the caudal part of the left flank.

**Figure 5:**
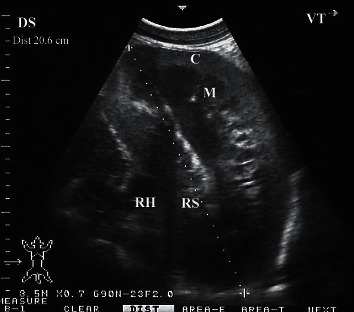
Ultrasonogram of a longitudinal section of the right kidney in a healthy camel. The image was taken from the upper right flank. RH = renal hilus; DS = dorsal; VT = ventral.

**Figure 6:**
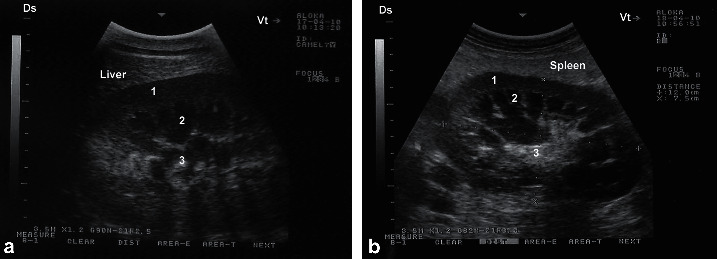
Ultrasonograms of a longitudinal section of the right (a) and left (b) kidneys in a healthy camel. Image (a) was taken from right 10th intercostal space through the so-called hepatic window. Image (b) was taken from the middle left flank through the so-called splenic window. 1, cortex; 2, medulla; 3, renal sinus' DS, dorsal; VT, ventral.

**Figure 7:**
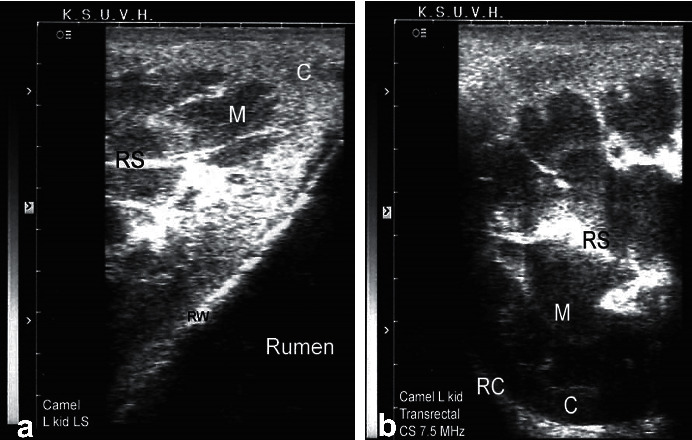
Transrectal ultrasonography of the left kidney in a healthy camel. The cranial pole of the left kidney is apparent (a). A cross-sectional view of the left kidney is also apparent (b). *C* = cortex; *M* = medulla; RS = renal sinus; RC = renal capsule; RW = rumen wall.

The right kidney can be visualized in camels at the level of the 10th and 11th intercostal spaces (ICS) and the upper right flank. The left kidney can be imaged from the caudal left flank (Figure 4). The differentiation between the renal cortex and medulla in both kidneys is visible in most cases. The renal cortex is relatively hyperechoic compared to the renal medulla and the renal sinus is hyperechogenic and more differentiated than the cortex and medulla. The right and left renal parenchyma are less echogenic than the neighbouring hepatic and splenic parenchyma, respectively. The medullary pyramids have a conic triangular appearance and are less echogenic than the remaining parenchyma. The renal hilus can be imaged when the transducer is placed in the paralumbar fossa and rotated about its longitudinal axis (Figure 5). However, the renal artery and vein and the ureter are difficult to be accurately identified. Ultrasonography via the so-called hepatic and splenic windows also results in good images of the right and left kidneys, respectively (Figure 6). When examined transrectally, the left kidney is accessible. In most camels, the entire left kidney is accessible and the cranial pole can be reached. The left kidney can also be easily imaged transrectally in a cross-sectional view (Figure 7) [18].

Table 1 shows the measurements of the right and left kidneys, including the distance to the body surface, the thickness of the cortex, medulla and renal sinus, and the vertical and horizontal diameters of both kidneys. The distance between the body surface and the left kidney is greater than that for the right kidney. The vertical diameter of both kidneys is significantly smaller than the horizontal diameter [18].

## 4. Ultrasound-Guided Renal Biopsy of the Right and Left Kidneys in Camels

In the camel, a biopsy of the right and/or left kidney is carried out in a sternal recumbency position. The fore-and-hind legs are tied by a rope near the carpal and hock joints, respectively. The distance between the 10th ICS to the flanks on both sides of the body is clipped and skin shaved. The shaved abdominal area was sterilized using standard surgical disinfection techniques. To obtain adequate restraint, camels are slightly sedated with xylazine (0.1 mg/kg·BW), and the region chosen for collecting hepatic or renal biopsy is infiltrated with 10 ml of 2% lidocaine hydrochloride. The kidneys are firstly scanned to determine the optimal biopsy sites. After the application of transmission gel to the transducer, the right and left kidneys are examined at the upper right and caudal left paralumbar fossa [21].

**Figure 8:**
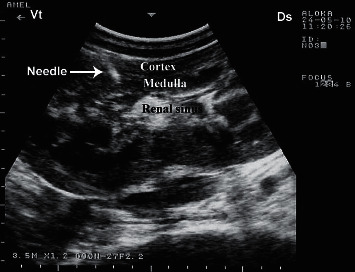
Renal biopsy in a camel. The needle is clearly visible within the renal cortex as a sharp bright line. Ds = dorsal; Vt = ventral.

Prior to biopsy, and under aseptic conditions, a small incision is made in the skin over the suggested biopsy site with the point of a scalpel blade. Using a free-hand technique, a 14 G × 150 mm spinal biopsy needle was used. The biopsy needle is then advanced through the skin incision and then under real-time ultrasound guidance toward the hepatic or renal parenchyma. During the biopsy of the kidneys, the advancement of the needle is halted when the tip of the needle is seen to penetrate the renal capsule. The needle is directed obliquely in an attempt to sample cortical tissue only and avoid the renal medulla, renal pelvis, and hilar and renal vessels. When the needle was considered to be in the correct position, the plain stylet is withdrawn and a notched part is inserted and advanced 1 cm into the renal cortex beyond the renal capsule. The needle can be identified on the ultrasound within the renal cortex as a sharp bright line (Figure 8), while the specimen was being obtained, thus confirming the location of the biopsy. Both the needle and forked stylet were then removed with a sample of renal tissue. Repeat passes were performed, if required, to obtain sufficient biopsy specimens. It is advisable to immediately scan the kidneys after biopsy to assess the presence of hematoma or active bleeding [21].

**Figure 9:**
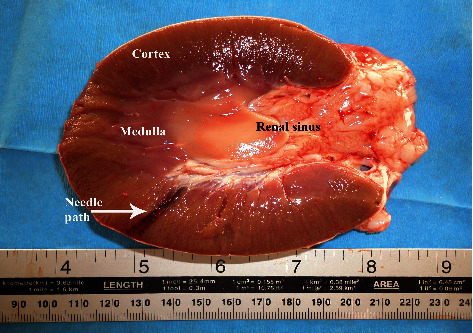
Under ultrasound guidance, renal biopsy needle path is usually easily identified within the renal cortex at post-mortem examination.

Low complications of renal biopsy are always achieved if the needle biopsy is advanced under ultrasound guidance, which provided more accurate localization of the needle in relation to the kidney and subsequent biopsy site in the renal cortex (Figure 9). Direct real-time ultrasound control allows the correction of the needle position at any moment during the biopsy procedure. Knowledge of the exact location of the needle in the cortex prevents deep penetration into the medulla [21].

## 5. Renal Disorders

### 5.1. Nephrolithiasis

**Figure 10:**
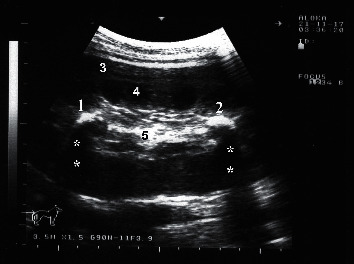
Nephrolithiasis (1 and 2) in a female camel with red urine for a 6-month period. 3, renal cortex; 4, renal medulla; 5, renal sinus. Stars point to acoustic shadowing under the calculi.

Urolithiasis is common as a subclinical disorder among ruminants raised in management systems where the ration is composed primarily of grain or where animals graze certain types of pasture. In these situations, 40–60% of the animals may form calculi in their urinary tract [37]. Urinary calculi (uroliths, nephrolith, bladder stone, and cystolith) are formed in either the calyces of the kidney or more commonly in the urinary bladder. In camels, small uroliths may enter the ureter or urethra and cause partial or complete obstruction of urine flow [38]. Urinary calculi are formed in males and females equally, but the bore (diameter) of the female urethra generally allows free passage of a calculus that may enter the urethra. Thus, obstructive urolithiasis is rare in the female and exclusive to the male animal. The majority of the calculi lodge in the sigmoid flexure of the penis, particularly in the distal segment. The composition of the calculi differs with geographic location. Silicate, phosphate, and calcium carbonate crystals have been observed [39]. High concentrate diets, improper calcium phosphorus balance, high silica or oxalate pasture, hypovitaminosis A, hypervitaminosis *D*, reduced water intake, and increased salts in drinking water are all possible factors for urolithiasis [40]. Calculus may be identified ultrasonographically in the kidneys of the camel as acoustic enhancement with distal acoustic shadowing (Figure 10).

### 5.2. Hydronephrosis

Chronic, unilateral, and ureteral obstructions are more likely to lead to hydronephrosis that is defined as a dilation of the renal pelvis with progressive atrophy of the renal parenchyma. Any urinary tract obstruction can lead to hydronephrosis, but the extent and duration of the obstruction are important in determining the severity of the renal lesion. Bilateral hydronephrosis may be caused by pelvic masses compressing the urethra or ureters.

**Figure 11:**
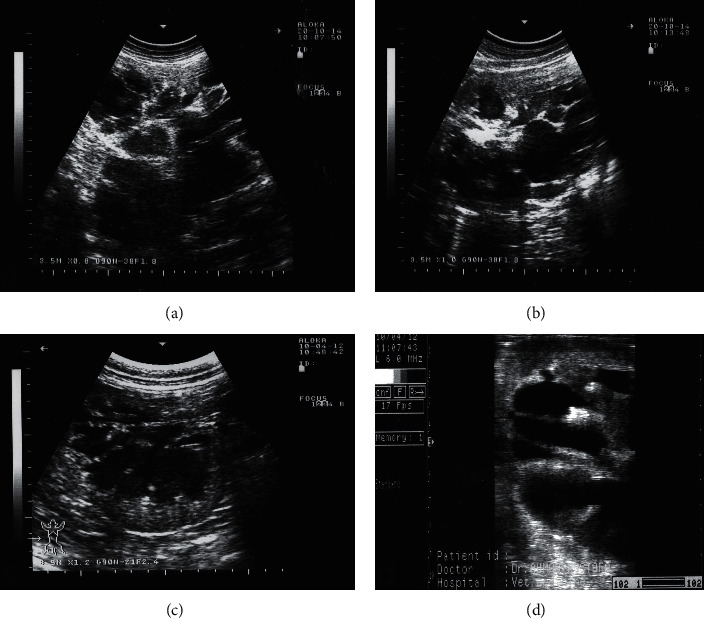
Bilateral hydronephrosis in two female camels with urinary calculi. Both the right (a) and left (b) kidneys were scanned transcutaneously. In the other case, the right kidney (c) was imaged transcutaneously and the left kidney (d) was imaged transcutaneously.

**Figure 12:**
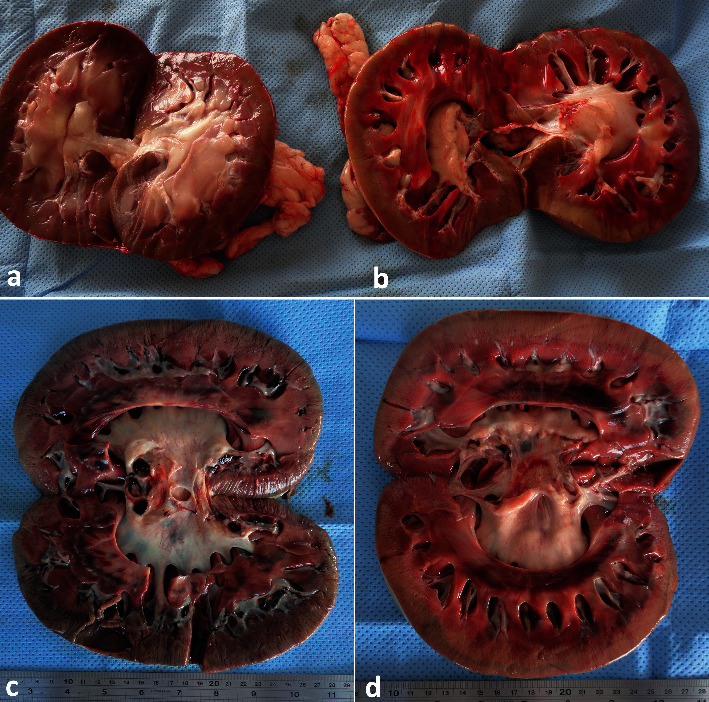
Unilateral hydronephrosis of the left kidney (b) in a female camel with pelvic abscessation detected postmortem compared to a healthy right kidney (a). Images (c) and (d) show bilateral hydronephrosis in a male camel with penile calculi.

The disease in ruminants can be a life-threatening, such as infected condition leading to pyelonephritis [41, 43]. To distinguish acute and chronic hydronephrosis, one may consider acute as hydronephrosis that, when corrected, allows full recovery of renal function. Conversely, chronic hydronephrosis is a situation in which the loss of function is irreversible even with the correction of the obstruction. A chronically dilated hydronephrotic system may be associated with compression of the papillae and thinning of the parenchyma; eventually, cortical atrophy progresses to the point at which only a thin rim of parenchyma is present [44]. Although diseased animals usually presented with some signs or symptoms, hydronephrosis can be an incidental finding encountered during the evaluation of an unrelated process. If unrecognized or left untreated, hydronephrosis can lead to loss of renal function and sepsis. Consequently, all animals found to have hydronephrosis should undergo a thorough evaluation [11]. In cases of bilateral hydronephrosis, transrectal ultrasonography showed a distended urinary bladder, anechoic fluid in the uterus, and hydronephrosis of the left kidney. By transcutaneous ultrasonography, the clinician can visibly scan and evaluate hydronephrosis of the right and/or left kidneys (Figure 11). The condition is usually identified postmortem as unilateral or bilateral affection (Figure 12).

### 5.3. Pyelonephritis and Renal Abscessations

**Figure 13:**
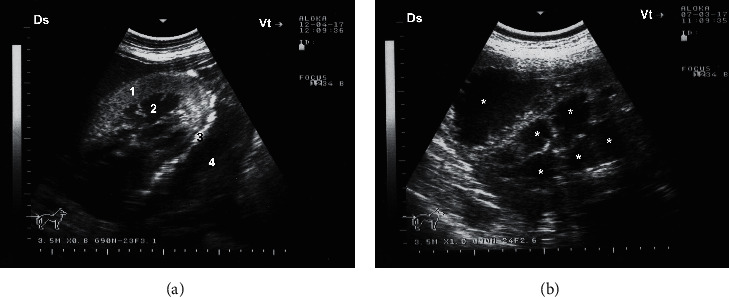
Transabdominal ultrasonographic examination of pyelonephritis in camels. Image (a) shows transcutaneous scanning of the right kidney with pyelonephritis in a male camel caused by *E. coli*. The renal capsule was imaged hyperechogenic with fibrin tags and the renal cortex appeared echogenic. A hypoechoic fluid was imaged surrounding the kidney. Image (b) shows transrectal view of unilateral pyelonephritis in a 6-year-old female camel caused by *Staphylococcus lugdunensis*. Heterogeneous contents were identified with multichambers containing echogenic fluid (stars). The cortex could not be differentiated from the medulla. 1, cortex; 2, medulla; 3, renal capsule; 4, perirenal abscessation.

**Figure 14:**
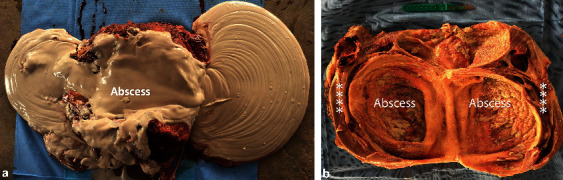
Gross pathological findings in a male camel with bilateral renal abscessation and chronic active pyelonephritis caused by *Escherichia coli*. Image (a) shows gross appearance of the left kidney and thick creamy pus evacuated from the kidney, weighting about 18 kg. Image (b) shows a longitudinal section through the affected kidney after complete evacuation of the large abscess revealed thickening and dilatation of the renal pelvis in relation to the renal abscess [30]. Stars point to the renal tissue.

**Figure 15:**
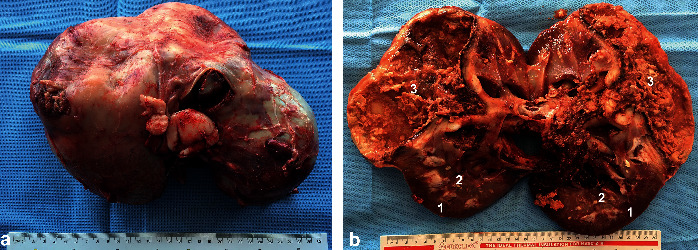
Gross pathological findings in a female dromedary camel with chronic suppurative pyelonephritis caused by *Staphylococcus* lugdunensis. Image (a) shows an enlarged abscessed kidney, while image (b) shows a longitudinal section of the affected kidney with corticomedullary abscess [31]. 1, cortex; 2, medulla; 3, abscess cavity.

In camels, renal pathologies such as abscess and pyelonephritis are rarely reported compared to other animals [39, 45]. Out of 121 slaughterhouse-obtained renal lesions in dromedary camels, only 7.43% were pyelonephritis [45]. Renal ultrasonography provides a precise, noninvasive, and fast technique for the evaluation and subsequent clinical decision-making of renal abscessation and chronic active pyelonephritis in dromedary camels. Recently, bilateral, and unilateral chronic active pyelonephritis together with renal abscessation caused by *E. coli* and *Staphylococcus lugdunensis* infection was reported in a 12-year-old male and 6-year-old female camel, respectively [30, 31]. In the male camel with bilateral pyelonephritis caused by *E. coli*, the transabdominal ultrasonographic examination of the right kidney revealed a hyperechogenic renal capsule with fibrin tags and echogenic renal cortex. A hypoechoic fluid was imaged surrounding the kidney from all sides and the kidney is floating within it. In the caudoventral left flank, the left kidney was imaged with a large volume of hypoechoic contents (Figure 13(a)). In the female camel with unilateral pyelonephritis caused by *Staphylococcus lugdunensis*, heterogeneous contents were identified with multichambers containing echogenic fluid. The cortex could not be differentiated from the medulla (Figure 13(b)). In both cases of pyelonephritis and abscessations, the diagnosis was verified postmortem (Figures 14 and 15).

**Figure 16:**
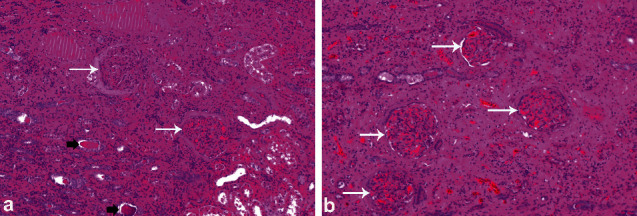
Histopathological findings of renal specimen of the right (a) and left (b) kidney in a camel with bilateral chronic active pyelonephritis showing renal cortical and medullary tissue with congested glomeruli (white arrows) and tubules with colloid casts and a fibrous and granulation tissue part at the periphery (black arrows). The interstitial tissue shows active and chronic cells infiltrate (H & E, ×100) [31].

**Figure 17:**
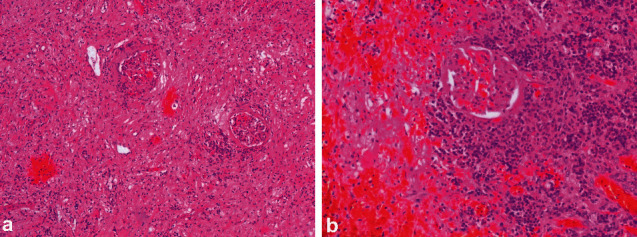
Histopathological section of the affected kidney showing congestion and periglomerular fibrosis, with atrophic tubules, hyaline, and RBCs casts. The interstitial tissue shows fibrosis, thick-walled blood vessels, and dense mixed inflammatory cell infiltrates. Area of necrosis with suppurative exudation is also seen (H & E, image *a* × 200; image *b* × 400) [30].

**Figure 18:**
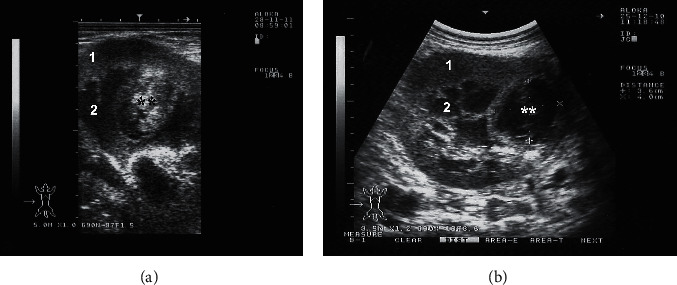
Transabdominal ultrasonographic examination of renal masses (*∗∗*) in 2 camels. Image (a) with echogenic mass was captured transrectally from the left kidney. Image (b) with hypoechoic mass in the right kidney was taken transcutaneously from the upper right paralumbar fossa. Both masses were confirmed to be abscesses through ultrasound-guided aspiration of pus. 1, cortex; 2, medulla.

In the male camel with bilateral chronic active pyelonephritis caused by *E. coli*, histopathological findings of both kidneys showed congested renal glomeruli and tubules with colloid casts and a fibrous and granulation tissue part at the periphery. The interstitial tissue showed active and chronic cells infiltrate (Figure 16) [31]. In the other female camel with unilateral pyelonephritis caused by *Staphylococcus lugdunensis*, histopathological findings of the affected kidney showed congestion and periglomerular fibrosis, with atrophic tubules, hyaline, and RBCs casts. The interstitial tissue showed fibrosis, thick-walled blood vessels, and dense mixed inflammatory cell infiltrates. Area of necrosis with suppurative exudation was also seen (Figure 17) [30]. Renal abscesses may be imaged localized as hyperechoic or hypoechoic masses (Figure 18).

### 5.4. Renal Neoplasia

**Figure 19:**
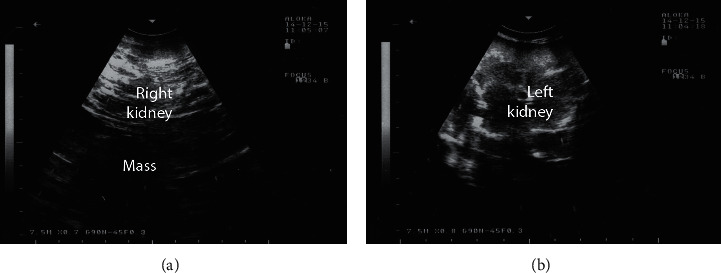
Transrectal ultrasonographic findings in a female camel with renal cell carcinoma of the right kidney. Image (a) shows a hypoechoic mass involving the right renal parenchyma, while image (b) shows the normal left kidney.

**Figure 20:**
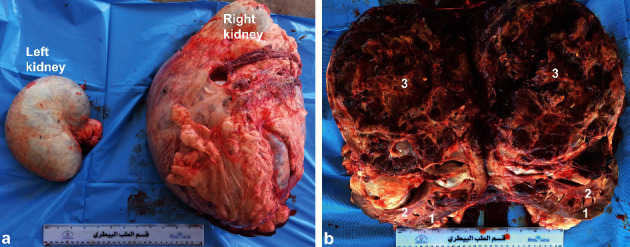
Postmortem findings in a female camel with renal cell carcinoma. Image (a) shows 18 Kg right kidney compared to 1.5 Kg left kidney. Image (b) shows cross section through the right kidney large, hemorrhagic, irregular shaped, and cavitated tumor [29]. 1, cortex; 2, medulla; 3, tumor mass.

**Figure 21:**
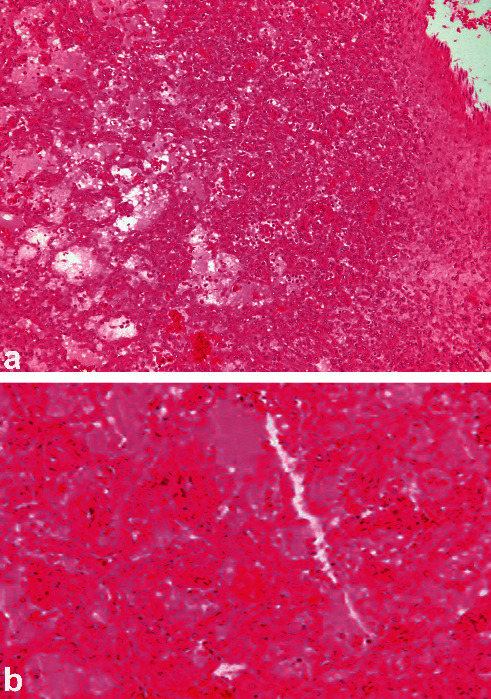
Renal cell carcinoma showing tubular differentiation with malignant epithelial lining and nuclear anaplasia (H&E, image *a* × 100; image *b* × 400) [29].

In dromedary camels, the most common tumors are squamous cell carcinoma, fibroma, adenocarcinoma, fibromyxosarcoma, leiomyoma, angiosarcoma, schwannoma, lipoma, microcystic adnexal carcinoma, renal cell carcinoma, Sertoli-Leydig cell tumour, and granulosa cell tumor [29, 46, 47]. In a case of confirmed right kidney renal cell carcinoma, transrectal ultrasonography revealed a caudally protruded, large, irregular shaped, hypoechoic, and cavitated mass involving the right renal parenchyma. However, the left kidney appeared subjectively normal (Figure 19). The ultrasound imaging is still to be verified at postmortem examination (Figure 20). Histological examination of the renal specimen revealed renal cell carcinoma showing tubular differentiation with malignant epithelial lining and nuclear anaplasia (Figure 21). No metastasis was found in other organs or even in the left kidney [29].

In conclusion, ultrasonography of the kidneys in dromedary camels is an important tool for imaging of the normal renal parenchyma as well as diagnosis, prognostication, and follow-up of treatment of kidney affections. This imaging modality can scan focal as well as diffuse renal lesions. Ultrasound-guided collection of renal specimens remains the final modality for confirmation of kidney lesions.

## Figures and Tables

**Table 1:** Dimensions (means ± SD) of the right and left kidneys imaged by ultrasound, from the upper right and caudal left paralumbar fossa in healthy camels [18].

Variable	Right kidney	Left kidney
Distance from body surface (cm)	1.8 ± 0.5	2.5 ± 0.6
Cortex (cm)	1.7 ± 0.6	1.7 ± 0.3
Medulla (cm)	2.7 ± 0.6	3.0 ± 1.0
Renal sinus (cm)	3.0 ± 0.7	3.6 ± 0.9
Vertical diameter (cm)	8.4 ± 1.4	9.8 ± 1.9
Horizontal diameter (cm)	18.1 ± 2.6	14.5 ± 3.0

## Data Availability

The data used to support the findings of this study are included within the article.
